# A cross-sectional survey of the knowledge, attitudes and practices regarding tuberculosis among general practitioners working in municipalities with and without asylum centres in eastern Norway

**DOI:** 10.1186/s12913-018-3792-4

**Published:** 2018-12-20

**Authors:** Oddvar Aadnanes, Selina Wallis, Ingunn Harstad

**Affiliations:** 1 Present Address: Legehuset Nova, Torggata 1, N-2317 Hamar, Norway; 2 Department of Public Health and Nursing, Faculty of Medicine and Health Sciences, Norwegian University of Science and Technology, 7489 Trondheim, NO Norway; 3 Public Health Institute, John Moores University, Liverpool, UK; 4 Department of Pulmonary Medicine, St Olavs University Hospital, Po Box3250 Sluppen, N-7006 Trondheim, Norway

**Keywords:** Tuberculosis (TB), Knowledge, attitudes and practice (KAP), Physicians, Norway

## Abstract

**Background:**

The number of tuberculosis (TB) cases in Norway is increasing due to immigration from countries with high TB prevalence and few studies have been conducted on general practitioners’ (GPs) knowledge of TB in low incidence countries. The main purpose of this study was to explore knowledge, attitudes and practices of TB among Norwegian GPs using a modified Knowledge Attitude Practice (KAP) survey template.

**Methods:**

A cross-sectional survey of 30 questions was distributed by email using SurveyMonkey to GPs working in municipalities either with or without an asylum reception centre in Eastern Norway (GPwAS or GPw/oAS). The questionnaire assessed demographic data and had 14 questions on TB knowledge and 7 questions on attitudes and practices. Descriptive and inferential analysis of the data was carried out using SPSS 18.

**Results:**

One hundred ninety five GPs responded and 42% worked in a municipality with an asylum reception centre. There was no significant difference between the two GP groups in relation to demographic variables (all *p*-values > 0.2). GPwAS were more experienced in diagnosing TB patients compared to GPw/oAS (63.4% vs 44.2%, *p* = 0.008). There was no significant differences in participation in TB training between the two groups (8.5% vs 7.6%, *p* = 0.71). The majority of GPs (69%) did not consider TB as a major public health threat and misconceptions of TB epidemiology were identified**.** Overall, 97 (49.7%) GPs had good TB knowledge level and good TB knowledge level was associated with experience in diagnosing TB patients (*p* = 0.001) and recent TB training (*p* = 0.015).

**Conclusion:**

Gaps in TB knowledge and awareness among GPs in Norway need to be addressed if GPs are to be more involved in TB management and prevention in the future. TB training had an effect on the GPs knowledge level and GPwAS had more experience with TB patients but our survey revealed no major differences in KAP between GPwAS and GPw/oAS.

**Electronic supplementary material:**

The online version of this article (10.1186/s12913-018-3792-4) contains supplementary material, which is available to authorized users.

## Background

Tuberculosis (TB) is a major global health threat. More than one-third of the world’s population is infected and 1.7 million people died from TB in 2016 [1]. Worldwide, the TB incidence rates have slowly declined since the year 2000 [1], but there are still major challenges associated with TB diagnosis, treatment success rate, patient compliance and multidrug-­-resistant tuberculosis (MDR-­-TB). In order to meet these challenges, the World Health Organization has suggested the goal of eliminating TB by 2050 [2, 3] where elimination of TB is defined as less than one TB case per million population per year. The majority of the European countries are low incidence countries, defined as less than 10 TB cases per one hundred thousand. However, the number of TB cases is increasing among foreign-born citizens due to immigration. Therefore, in order to align with the vision of TB elimination, it is important to maintain knowledge and awareness of TB and sustain a focus on groups at risk as well as ensure strong cooperation with related stakeholders [4].

Norway is a low-incidence country with a population of 5.2 million and has historically had a strong focus on TB prevention and control. TB epidemiology in Norway is characterized by a low rate of transmission within the general population, occasional outbreaks and the majority of cases relates to cross-border migration and progression of latent TB. In 2015, the incidence of TB was 6 per 100′000, with marked differences in TB incidence between population groups and altogether 318 cases were reported [5]. A low incidence of TB among native-­-born Norwegians (0.6 per 100,000) in 2015 indicates that GPs in Norway may have infrequent experience of diagnosing TB. Approximately 90% of the cases in Norway are infected outside the country and today the majority of TB cases are detected among asylum seekers as shown in Table 1. Patients presenting with TB are born in countries with a high prevalence of TB and active TB disease is mainly due to the reactivation of latent TB [5]. Statistically, 5-­-15% of persons with latent TB will develop active TB during their lifetime [1] and the shift in TB epidemiology due to immigration has led to a growing pool of latent TB in Norway. Surveys confirm that immigrants in Norway have an increased risk of developing TB many years after their arrival [6] and have revealed that asylum seekers are a group of immigrants with a high risk of developing TB [7, 8].

**Table 1:** Background, number and proportion of foreign-­-born TB cases in Norway 2013–2017

Background	2013		2014		2015		2016		2017	
	n	%	n	%	n	%	n	%	n	%
Asylum seekers	194	55.1	172	57.0	157	56.3	138	52.7	101	43.7
Family reunion	47	13.4	61	20.2	46	16.5	56	21.4	52	22.5
Immigrant	56	15.9	33	10.9	43	15.4	38	14.5	42	18.2
Temporary residence	39	11.1	18	6.0	17	6.1	15	5.7	19	8.2
Other	16	4.5	15	3.9	16	5.7	15	5.7	17	7.4
Total	352	100	302	100	279	100	262	100	231	100

The Norwegian Institute of Public Health, 2014–2017

The Norwegian Directorate of Immigration (UDI) is responsible for the organization of accommodation for asylum seekers in collective open reception centres throughout the country. At the time of our study, there were 110 asylum reception centers accommodating 14′400 asylum seekers [9]. These asylum seekers originated from 110 different countries and a major percentage came from countries with a high prevalence of TB such as Eritrea, Somalia, Sudan, Afghanistan and Ethiopia. The Municipality Medical Officers have by Norwegian law the overall responsibility for implementation of the TB control strategy in Norway. Specialists in the secondary care handle active TB disease while TB screening is organized by the Municipality Medical Officers and conducted by public health nurses. The increasing number of latent TB cases has led to a growing tendency to involve GPs in risk assessment due to capacity and resource constraints in secondary health care. This is challenged by GPs lack of awareness and clinical experience due to the low TB incidence within the native Norwegian population. Multiple interacting risk factors make the TB epidemiology complex [10] and in low-burden countries, TB control requires various approaches in order to be effective among population groups with different TB prevalence [11]. Today, the strategy for the involvement of GPs in Norway is unclear, and to be able to eliminate TB it is essential that the GPs have the knowledge and skills to undertake early detection, preventive measures and a thorough understanding of people at risk. There are only a few studies from other European countries exploring primary care physician’s knowledge and handling of TB and no published studies available from Norway. This study was designed to explore knowledge, attitudes, and practices (KAP) on TB among general practitioners (GPs) working in municipalities in Eastern Norway. The secondary aim was to explore any differences in KAP between GPs that work in municipalities with an asylum reception centre (GPwAS) compared to GPs working in municipalities without an asylum reception center (GPw/oAS).

## Methods

### Study design and data collection

Eastern Norway is the most populated area in Norway with around 50% of the population and 41 of the total 100 municipalities with asylum centers were located in this region as of 2014 [9]. Our target area was selected due to the majority (60%) of Norway’s total 378 TB cases being detected here in 2012 [12]. This cross-sectional survey took place from 5th of November 2014 until 4th of December 2014 and prior to our study 2′126 GPs were employed in the target area, which accounted for 39% of all GPs working within the regular GP scheme in Norway [13].

GPs working within the regular GP scheme in the target area were eligible independent of their length of employment in the selected municipalities. The GPs name, address and phone number were available from the Norwegian Health Economic Administration (HELFO). GPs working privately or outside the GP scheme in the same municipalities were excluded from the survey. GP participants were randomly selected from the Norwegian Health Economic Administration using online software [14]. The recruitment of the GP participants started 3 months prior to the survey start since email address to selected GP participants had to be collected from Medical Officers in the municipalities, direct phone contact with GPs workplace or directly to the selected participants. The participants were divided into two strata based on the existence of an asylum reception centre in their municipality. Municipalities with asylum reception centres were labeled GPwAS. Municipalities without asylum reception centres were labeled GPw/oAS. The ratio between GPwAS and GPw/oAS was about 1 to 3. A total of 689 GPs worked in GPwAS [13]. Calculation using statistical software gave a minimum sample of 247 [15] from each of the two strata. Adjusting for non-responders 721 eligible GPs received an email invitation and could access the questionnaire online through SurveyMonkey.

### Questionnaire

The questionnaire consisting of 30 questions was based on a KAP survey template [16] (Additional files 1 & 2). This template was modified and adapted to the Norwegian setting, language and national TB guidelines and then validated by TB experts and Municipality Medical Officers. Finally, the questionnaire was piloted among GPs working in the target area. The questionnaire assessed demographic information, the number of TB patients seen in the last 3 years, participation in TB training within the last 12 months, and KAP data on diagnosis, treatment and infection control based on TB guidelines developed by the Norwegian Institute of Public Health [17]. There were 14 questions related to TB knowledge and 7 questions regarding TB attitudes and practices. The questionnaire is presented in the appendix. The participants were instructed to select a certain number of responses to each question and the online survey settings restricted the participants from providing additional responses.

### Data analysis

Response data on the questionnaire provided in SurveyMonkey was imported into SPSS 18 and recoded. The knowledge score was calculated from a total of 14 points. The mean score for both groups was compared and a score above the median value was considered good knowledge [18, 19]. All correct responses were determined using the national TB guidelines [17]. Chi-­-squared analysis was used to compare proportions of good TB knowledge between the two GP strata. Analysis of variance (ANOVA) was used to identify differences in mean knowledge between the two GP strata as well as comparing the knowledge score and their participation in relevant training. ANOVA was also used when comparing knowledge score and working experience. Chi-­-squared analysis was used to compare TB knowledge level and variables like participation in recent TB training, experience with TB patients and specialization. Significance was considered at a p-­-value < 0.05. Descriptive statistics were described for the seven questions related to attitudes and practices. An ANOVA test was conducted to identify significant differences in perception of TB as a health threat in the two GP strata.

### Ethical considerations

Ethical approval was received from the University of Liverpool. The Regional Committee for Medical Research Ethics of Southern Norway (REC) considered the study to be outside the remit of the Health Research Act and therefore the study could be implemented without approval of REC. Information about the study and informed consent was provided in writing before the participants were able to access the online survey questionnaire. Confidentiality and anonymity were maintained through the settings of the online survey.

## Results

The online survey questionnaire was sent out to 721 GPs and 210 gave informed consent and the survey was completed by 195 of whom 42% was GPwAS. Due to settings limitations, the respondents were not able to submit the questionnaire unless answering all the questions and the respondents not answering the questions were considered as non-responders. The total response rate was 27%. Men represented 55% of the participants and age distribution showed that 35% of the GPs were less than 40 years old, 48% between the age 41–60 and 17% of the GPs were older than 60 years. The size of the patient lists varied and 40% reported a list between 901 and 1200 patients, 36% had a list of more than 1200 patients while 23% had less than 900 patients. A majority of the participants (64%) were GP specialists and 58% of the GPs had worked for more than 10 years in general practice. There were no significant differences in demographic variables or years of GP experience between GPwAS and GPw/oAS as shown in Table 2.

**Table 2:** Demographic characteristics of the GP participants

	Proximity to asylum reception centre				*P*-value	Total	
	Yes	%	No	%		n	%
General Practitioners	82	42.1	113	57.9		195	100
Sex							
Male	44	53.7	63	55.8	0.772	107	54.9
Female	38	46.3	50	44.2		88	45.1
Age in years							
< 30	4	4.9	1	0.9	0.309	5	2.6
31–40	26	31.7	37	32.7		63	32.3
41–50	17	20.7	31	27.4		48	24.6
51–60	18	22.0	27	23.9		45	23.1
> 60	17	21.7	17	15.0		34	17.4
GP specialist							
Yes	50	61.0	75	66.4	0.438	125	64.1
No	32	39.0	38	33.6		70	35.9
Patient list							
< 500	2	2.4	5	4.4	0.547	7	3.6
501–900	20	24.4	19	16.8		39	20.0
901–1200	32	39.0	46	40.7		78	40.0
1201–1500	20	24.4	35	31.0		55	28.2
> 1500	8	9.8	8	7.1		16	8.2
GP experience in years							
< 1	2	2.4	5	4.4	0.269	7	3.6
1–4	17	20.7	17	15.0		34	17.4
5–9	19	23.2	22	19.5		41	21.0
10–14	8	9.8	23	20.4		31	15.9
> 15	26	31.7	46	40.7		82	42.1

### Participation in TB training and experience with TB case detection

Less than 8 % (7.7%) of the 195 GPs had attended TB training in the past 12 months and these GPs were equally distributed among GPwAS and GPw/oAS (Table 3). About half of all the GPs (48%) had no experience with TB patients the last three years, while 34% had diagnosed one or two patients with TB and 11% had diagnosed three or four patients. Experience with diagnosing more than five patients was reported by 7% of the respondents. Analysis showed that 63% of the GPwAS had diagnosed one or more TB patients while only 44% of the GPw/oAS and GPwAS had significantly more experience in diagnosing TB patients compared to GPw/oAS (Table 3).

**Table 3:** Proximity to asylum reception centre and relation to recent TB training and diagnosed TB patients last three years

	Proximity to asylum reception centre				*P*-value	Total	
	Yes	%	No	%		n	%
Participation in recent TB training							
Yes	7	8.5	8	7.1	0.707	15	7.7
No	75	91.5	105	92.9		180	92.3
Total	82	100.0	113	100.0		195	100.0
Diagnosed TB patients							
None	30	36.6	63	55.8	0.008	93	47.7
One or more	52	63.4	50	44.2		102	52.3
Total	82	100.0	113	100.0		195	100.0

### TB knowledge

Overall, the participants answered 64% of the questions correctly. Out of the highest possible score of 14 points, the mean knowledge score was 8.3 ± 2.0. The median knowledge score was 8 ranging from 2 to 13. A score above 8 was considered good TB knowledge and 50.3% of the GPs had a score of 9 or higher on the 14 selected questions related to TB knowledge.

Analysis on demographic variables like sex, age, specialty in general practice, patient list size and years of experience showed no significant difference in mean TB knowledge score between GPwAS and GPw/oAS (Table 4), as well as no significant difference in TB knowledge level between GPwAs and GPw/oAS (Table 5).

**Table 4:** Distribution of mean TB knowledge score and knowledge level by GPs proximity to Asylum Reception Centre

	Proximity to Asylum Reception Centre					
	Yes			No		
	Mean TB Knowledge score X ± SD	Knowledge Level (n)		Mean TB Knowledge score X ± SD	Knowledge Level (n)	
		Good	Poor		Good	Poor
Sex						
Male	8.48 ± 2.27	22	22	8.13 ± 1.90	30	33
Female	8.34 ± 2.08	20	18	8.26 ± 1.98	25	25
Age in years						
< 30	8.75 ± 2.98	2	2	7.00	0	1
31–40	8.31 ± 2.33	13	13	8.62 ± 1.95	23	14
41–50	9.29 ± 1.76	12	5	8.32 ± 1.70	17	14
51–60	8.11 ± 1.88	6	12	7.81 ± 1.75	9	18
> 60	7.94 ± 2.34	9	8	7.65 ± 2.40	6	11
GP specialist						
Yes	8.28 ± 2.06	23	27	7.76 ± 1.94	29	46
No	8.63 ± 2.35	19	13	9.02 ± 1.58	26	12
Patient list						
< 500	10.5 ± 2.12	2	0	8.8 ± 1.64	4	1
501–900	8.5 ± 2.62	12	8	8.84 ± 1.53	12	7
901–1200	8.28 ± 2.31	17	15	8.19 ± 2.11	24	22
1201–1500	8.30 ± 1.78	7	13	7.80 ± 1.79	13	22
> 1500	8.50 ± 1.19	4	4	7.88 ± 2.23	2	6
Years of GP experience						
< 1	8.50 ± 4.95	1	1	8.60 ± 2.07	4	1
1–4	8.58 ± 2.06	10	7	9.35 ± 1.57	13	4
5–9	8.84 ± 1.86	10	9	7.72 ± 1.90	10	12
10–14	8.00 ± 3.11	4	4	8.34 ± 1.87	12	11
≥ 15	8.19 ± 2.08	17	19	7.84 ± 1.93	16	30

**Table 5:** Proximity to Asylum Reception Centre and TB Knowledge Level

	Proximity to Asylum Reception Centre				*P*-Value	Total	
	Yes	%	No	%		N	%
TB knowledge level							
Good	42	51.2	55	48.6	0.725	97	49.7
Poor	40	48.8	58	51.3		98	50.3
Total	82	100.0	113	100.0		195	100.0

There was a negative association between good TB knowledge level and GP specialization but also evident that specialists had diagnosed less TB patients compared to non-specialists among both GPwAS and GPw/oAS. Good TB knowledge level was positively associated with GPs that had attended TB training and GPs that had experience in diagnosing one or more TB patients for the last three years (Table 6).

**Table 6:** TB knowledge level related to specialization, training and experience with TB patients

	TB Knowledge level				*P*-Value	Total	
	Good		Poor				
	n	%	n	%		n	%
GP Specialist							
Yes	52	53.6	73	74.5	0.002	125	64.1
No	45	46.4	25	25.5		70	35.9
Total	97	100.0	98	100.0		195	100.0
TB Training							
Yes	12	12.4	3	3.1	0.015	15	7.7
No	85	87.6	95	96.9		180	92.3
Total	97	100.0	98	100.0		195	100.0
Experience with TB patients							
Yes	62	63.9	40	40.8	0.001	102	52.3
No	35	36.1	58	59.2		93	47.7
Total	97	100.0	98	100.0		195	100.0

**Fig. 1:**
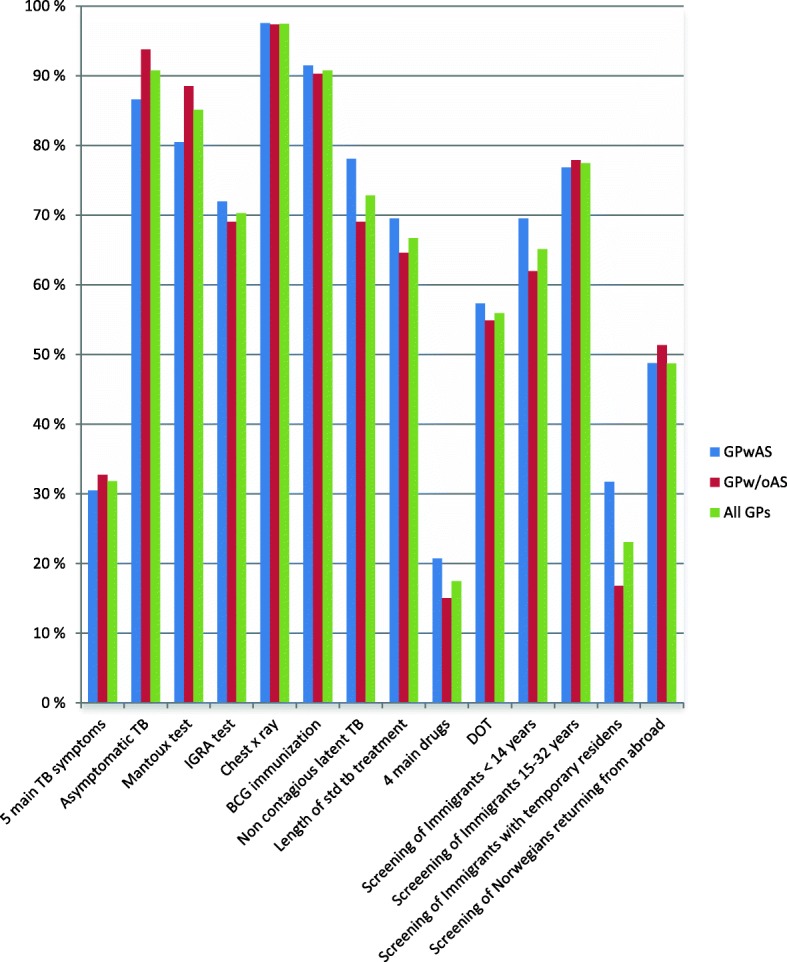
Distribution of correct answers on questions related to TB knowledge

**Fig. 2:**
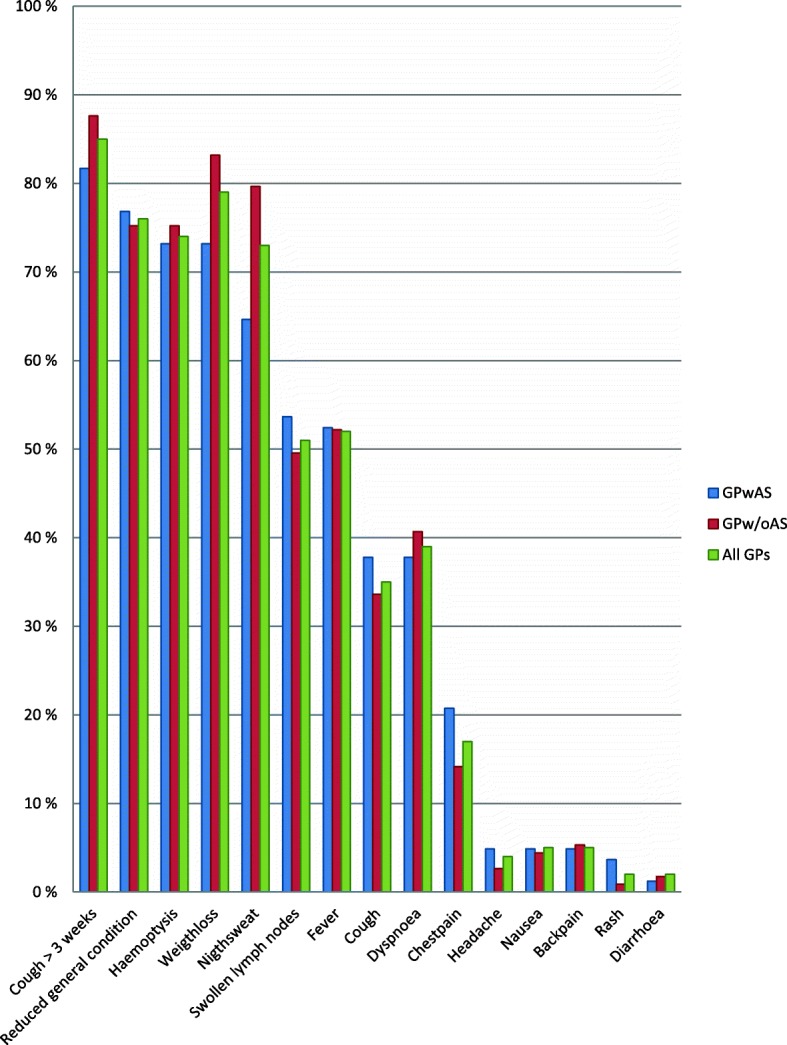
Distribution of answers related to classical symptoms of active pulmonary TB

As presented in Fig. 1, over 90% of the GPs knew that a negative chest x-ray does not exclude TB infection, BCG vaccination does not ensure 100% protection against TB and that a person infected with TB can be asymptomatic and go through life without being sick. While 70–85% of the GPs responded that a positive Mantoux test does not mean definite TB disease, latent TB is non-infectious and that they would refer patients with positive IGRA to specialists. In Norway, the minimum duration of treatment for active TB is six months which was correctly identified by 61% of the GPs. 44% of the GPs had never heard about Directly Observed Therapy (DOT), the standard treatment strategy in Norway and recommended TB control strategy by the WHO. Questions related to the five main symptoms of TB, the four standard drugs, and screening of immigrants with temporary residence had the lowest score. The classical symptoms of active pulmonary TB are according to the Norwegian Institute of Public Health a cough for more than three weeks, weight loss, reduced general condition, night sweats and fever. Less than one-third of the GPs identified these classical TB symptoms (Fig. 1). They associated haemoptysis more frequently with TB than night sweats and fever. Haemoptysis is a rare symptom of pulmonary TB in Norway. Some respondents indicated wrongly that diarrhoea, nausea, rash or headache are main symptoms of pulmonary TB (Fig. 2). The four drugs that are part of the standard treatment for TB in Norway were identified by 17% of the GPs while 21% did not identify any correct drug. There were four questions related to the screening of immigrants from high prevalence countries and ethnic Norwegians returning from long-term stay abroad and intend to work in health institutions or the school system. Sixty percent of the GPs had two or less correct answers and 32% had three correct answers on these questions, while only 8 % of the GPs were familiar with all four correct answers.

### Attitudes and practices regarding TB detection and control

The majority of the GPs (64%) did not consider TB as a major health threat in Norway today and there was no significant difference in perception of health threat between GPwAS and GPw/oAS. The GPs responded that immigrants, people living with HIV/AIDS and family members of a confirmed case are groups most at risk for developing TB and a great part of the GPs also responded that addicts, homeless persons and healthcare professionals are at greater risk developing TB in Norway today (Table 7). On questions related to TB transmission, almost all GPs (98%) correctly identified that TB is transmitted by air droplets when a person with TB coughs or sneezes but at the same time a large proportion of the GPs reported incorrectly that TB could infect a person through sharing dishes (28%) and eating from the same plate (20%).

**Table 7:** Attitudes and practices related to TB among the 195 GPs included in the survey

	GPwAS		GPw/oAS		Total	
	n	%	n	%	n	%
Who are the persons most likely to become infected with TB in Norway? (Multiple answers)						
Homeless persons	22	26.8	41	36.3	63	32.3
Children less than 5 years	2	2.4	5	4.4	7	3.6
Senior citizens	10	12.2	14	12.4	24	12.3
People living with HIV/AIDS	56	68.3	87	77.0	143	73,3
Health care workers returning from assignments abroad	30	36.6	31	27.4	61	31.2
Health care workers treating a confirmed case	27	32.9	30	26.6	57	29.2
Immigrants	64	78.1	87	77.0	151	77.4
Family members of a confirmed case	56	68.3	75	66.4	131	67.2
Prison inmates	6	7.3	14	12.4	20	10.3
Drug users	34	41.5	55	48.7	89	45.6
Is TB a major public health threat in Norway?						
Yes. TB is already more than just a major threat	4	4.9	4	3.5	8	4.1
Yes. TB poses a serious threat to Norway	21	25.6	31	27.4	52	26.7
No. TB are well controlled so there is no major concern	52	63.4	69	61.1	121	62.0
No. TB is not even a small threat at this time	2	2.4	3	2.7	5	2.6
Not sure	3	3,7	6	5.3	9	4.6
How can a person become infected with TB? (Multiple answers)						
Through handshakes	3	3.7	7	6.2	10	5.1
Through the air when a person with TB coughs	79	96.3	112	99.1	191	97.8
Through sharing dishes	18	22.0	36	31.9	54	27.7
Through eating from the same plate	12	14.6	27	23.9	39	20.0
Through contact with blood	9	11.0	7	6.2	16	8.2
Through food and water	4	4.9	10	8.9	14	7.2
Through touching items in public	3	3.7	6	5.3	9	4.2
Through unprotected sex	3	3.7	3	2.7	6	3.1
Not sure	3	3,7	1	0.9	4	2.1
Under what circumstances are health education messages on TB given to patients? (Multiple answers)						
World TB day	1	1.2	0	0	1	0.5
BCG immunization	10	12,2	9	8.0	19	9.7
General health promotion	12	14.6	14	12.4	26	13.3
Suspected or confirmed cases	24	29.3	26	23.0	50	25.4
Suspected cases and their families in a clinical setting	21	25.6	30	26.6	51	26.2
Confirmed cases and their families in clinical or community setting	3	3.7	5	4.4	8	4.1
Health education on TB in general not provided	41	50.0	60	53.1	101	51.8
Others	8	9.8	8	7.1	16	8.2
What is the primary diagnostic test that is usually requested to confirm or rule out a case of active pulmonary TB?						
IGRA test	34	41.5	39	34.5	73	37.4
Chest X ray	13	15.9	19	16.8	32	16.4
Mantoux test	8	9.8	11	9.7	19	9.7
Sputum smear microscopy / culture	21	25.6	36	31.9	57	29.2
Blood culture	0	0	0	0	0	0
Not sure	6	7.3	8	7.1	14	7.2
When can a TB patient be considered as noninfectious?						
Received adequate TB treatment for minimum 2 weeks	13	15.9	25	22.1	38	19.5
Negative chest X-ray	1	1.2	2	1.8	3	1.5
No cough	6	7.3	6	5.3	12	6.2
Completed the whole treatment	30	36.6	38	33.6	68	34.9
Conversion of IGRA test from positive to negative	14	17.1	12	10.6	26	13.3
Not sure	18	22.0	30	26.6	48	24.6
What is your role as GP when one of your patients is treated for TB?						
To be kept informed about the ongoing treatment and when appropriate be involved in the clinical monitoring of the patient under supervision by specialist	66	80.5	88	77.9	154	79,0
No role. The responsibility lays with the specialist, TB coordinator and medical officers in the municipality	8	9.8	7	6.2	15	7.7
Not sure	8	9.8	18	15.9	26	13.3

Sputum smear analysis was identified correctly by 29% of the GPs as the primary diagnostic test for active TB while an even larger proportion of the GPs (38%) answered incorrectly that IGRA test is the primary diagnostic test used in either confirming or ruling out active TB.

Twenty percent knew correctly that a patient that had received correct treatment for pulmonary tuberculosis for two weeks is considered as non-infectious. However, 35% responded incorrectly that the patient had to complete the whole treatment in order to be non-infectious whereas 25% were not sure and 13% responded the need for IGRA test conversion. The majority of GPs did not provide health education on TB. However, 26% percent had experience in providing health education in a clinical setting related to a suspected or confirmed TB case and 13% responded they had provided health education on TB as part of their general health promotion. Only 10 % reported experience in providing health education related to BCG immunization.

The large majority of the GPs (79%) would like to have an active role in treatment and follow-up of their TB patients while 8 % responded that they felt they had no role. Uncertainty about the GP role was detected among 13% of the respondents and uncertainty about the GP role was associated with lower TB knowledge score. There were no significant differences in perception of role when comparing GPwAS and GPw/oAS.

## Discussion

In this study, less than 50% of the GPs had a score above 8 out of a total of 14 points and considered as good knowledge of TB, which is a concern, along with evidence of gaps in knowledge covering several areas like symptoms, diagnoses, transmission, treatment, and screening. More specific areas of concern were the fact that less than one-third of the GPs identified the five main symptoms of pulmonary tuberculosis, and between 20 and 30% indicated that a person can get infected with TB through sharing dishes and eating from the same plate. This may indicate a superficial understanding of when to suspect TB infection and limited ability to provide correct information in communication with patients, family members and people at risk. This also raises concerns about GPs awareness regarding the implementation of simple control measures like wearing masks as transmission control. Previous studies from South Africa and Ethiopia have identified good knowledge in infection control as a predictor of good practice [20, 21], while other studies have highlighted that major gaps in TB knowledge could result in uncertainties and non-stringent TB management [22]. The GPs had in general low knowledge about standard TB treatment, drugs and DOT treatment. This is likely due to the fact that secondary care specialists are responsible for the final diagnosis and treatment but at the same time, most cases of TB and latent TB will be referred to GPs for regular blood test control during the treatment. Our survey indicates that GPs knowledge level of TB is insufficient if they are to be more frequently involved in the follow-up of TB cases.

In October 2014, The Norwegian Institute of Public Health [17] presented a revised and simplified version of the TB flow chart on TB screening and case detection. IGRA testing is now the preferred test compared to Mantoux testing and according to NIPH guidelines, the GPs are supposed to be informed if one of their patients has a positive IGRA test. The GPs had lower scores on questions related to IGRA testing compared to Mantoux testing, which may indicate that the GPs are less familiar with the test and how to interpret the results.

In our survey, 60% of the GPs had two or less correct answers on four questions related to TB screening which shows a high degree of uncertainty among GPs related to screening. This could result in TB cases not being referred to specialists or diagnosed and cases of latent TB not being detected. This aligns to previous studies, which have identified weaknesses in the follow-up of TB screening of asylum seekers at all levels of care in Norway [7]. Previous research has emphasized the importance of screening and treatment of latent TB in order to reduce the TB incidence among immigrants [23, 24] and according to World Health Organization [3], such actions will support the ambitious targets of the End TB Strategy of a 90% reduction in TB.

In our study, there were no significant differences in knowledge between GPwAS and GPw/oAS. However, good TB knowledge level was positively associated with GPs that had attended TB training and GPs that had experience in diagnosing TB patients. There is little research available on how experience with TB among primary health care providers (PHCP) in low incidence countries influences on early detection and TB management. At the same time, the low number of TB patients and irregular involvement in TB diagnosis might hinder the possible positive association between years of experience and performance effectiveness. This is similar to findings revealed in studies related to other complex health services GPs are involved in [25]. At the same time other studies have concluded that training needs to be combined with guidelines implementation or introduction of collaborative care models in order to have an impact on GPs behavior [26]. Lack of awareness, as well as limited numbers of available training courses or workshops on TB, could be the reasons why only a few GPs had participated in TB courses. TB related topics are sometimes included as a minor part of courses on infectious diseases and GPs have the opportunity to attend a National TB conference held every second year. While in the United Kingdom (UK) there is an hour online training course aimed at improving the GPs strategies on diagnosis and management of TB, and online learning courses have proven to be beneficial in several previous studies both in Norway and elsewhere [27, 28].

Our study indicates that GPs perceive TB as a low health threat and GPs were not frequently involved in TB health promotion. The GPs also had a tendency of overestimating the risk of TB among certain population groups less likely to become infected with TB today. This might indicate that TB awareness among GPs in Norway is generally low and that GPs are not familiar with the shift in TB epidemiology. The rate of TB differs between the European countries and is explained by the difference of geographical origin of migrants [29]. It is evident that TB is a greater public health problem among homeless people, prisoners and drug users in countries like the UK and the Netherlands compared to Norway [30]. These are population groups that are challenging in relation to follow-up and adherence to treatment but few TB cases in Norway are categorized within these subpopulation groups. However, experience with educational interventions to promote TB screening has emphasized the importance of reaching out to all groups at risk and avoid the pitfalls of ethnic profiling [31]. Low TB awareness could lead to clinical mistakes in diagnosing and treating TB and increases the risk of TB outbreaks [32]. In our study only 29% identified sputum smear analysis as the primary diagnostic test in order to confirm or rule out active pulmonary TB, and only 20% knew that a TB patient needs minimum 2 weeks of adequate treatment in order to be considered as noninfectious. TB is a rare disease in Norway but there is a growing pool of latent TB. Raised awareness and improved capability of early diagnosis will probably be a necessary contribution from primary care to TB control in the future. In the UK, TB cohort audit (TBCA) has been recommended in the National Institute for Health and Care Excellence (NICE) guidance since 2012 [33]. TBCA has been used as a tool in order to strengthen cross-professional collaboration and health professionals have reported changes in their practice and enhanced understanding of the different roles of the stakeholders involved in TB care [34].

### Strengths and limitations

Our survey had lower response rates than expected and did not meet the calculated sample size of 247 participants. However, with a response rate of 27% and 195 respondents, it was still enough to assess statistical significance and the sample size was comparable with other studies [35]. Unrewarded surveys among busy GPs with a long questionnaire and few respondents with special interest in the subject are at risk of low response rates and sampling bias might be likely so generalization of the results should be considered carefully [36, 37]. The majority of TB cases in Norway are detected in the target area of this study. Thus, if all municipalities nationwide had participated in our research the knowledge level might be even lower than that found in our survey. GPs that participated in this survey might also have been more interested in TB compared to other GPs, and therefore this study might actually overestimate the TB knowledge level among GPs in Norway. A previous TB study by Lobue [38] concluded that physicians that have treated six or more TB patients for the last two years were more knowledgeable about local guidelines.

The size of patient lists was quite equally distributed. However, the numbers of immigrants in the GPs lists was not accounted for and a higher number of immigrants might influence the GPs interest in TB and participation in studies like this or imply more experience in TB management. The number of colleagues in the general practices was not accounted for in our study but the TB knowledge level might be influenced by the number of colleagues sharing their experience with TB cases [39].

## Conclusion

This was the first cross-sectional survey assessing KAP on TB among GPs in Norway and will add to the limited numbers of studies performed in low incidence countries in Europe. Knowledge and awareness of TB among GPs in Norway is low and while asylum reception centres represent a cluster of TB and latent TB, the GPs working in these municipalities are not more likely to have received training or have better knowledge of preventive measures than other GPs. Today, specialists, TB coordinators and public health nurses are most involved in TB management while the role of the GPs is not yet defined. Our survey presents only a snapshot on KAP related to TB but the results could inform the process of defining the GPs role in TB management in the future.

## Additional files

Additional file 1: TB survey among Norwegian GPs survey questionnaire ENG: The survey questionnaire in English. (PDF 210 kb)

Additional file 2: TB survey among Norwegian GPs survey questionnaire NOR: The survey questionnaire in Norwegian. (PDF 208 kb)

## Abbreviations

ACSM: Advocacy, communication and social mobilization
AIDS: Acquired immunodeficiency syndrome
ANOVA: Analysis of variance
DOT: Directly observed treatment
GP: General practitioner
GPwAS: GPs that work in municipalities with an asylum reception centre
GPwoAS: GPs working in municipalities without an asylum reception centre
HIV: Human immunodeficiency virus
KAP: Knowledge, attitudes and practices
MDR-­-TB: Multidrug resistant tuberculosis
NICE: National Institute for health and care excellence
NIPH: Norwegian institute of public health
PHCP: Primary health care professional
TB: Tuberculosis
TBCA: TB cohort audit
UK: United Kingdom
WHO: World Health Organization
