# Supplementary material for: A cross-sectional survey of the knowledge, attitudes and practices regarding tuberculosis among general practitioners working in municipalities with and without asylum centres in eastern Norway
Source: BMC Health Serv Res. 2018 Dec 20;18:987. doi: 10.1186/s12913-018-3792-4 (PMC6302494; doi:10.1186/s12913-018-3792-4)
Supplement: Supplementary file 1 — TB survey among Norwegian GPs survey questionnaire ENG: The survey questionnaire in English. (PDF 210 kb) [file 12913_2018_3792_MOESM1_ESM.pdf]

Tuberculosis survey among Norwegian GPs, November 2014

Questionnaire information

You are hereby invited to participate in a survey assessing the knowledge, attitudes and practices of tuberculosis (TB) among general practitioners (GPs) in Eastern Norway. However, before you decide if you wish to participate, it is important that you know why the research is being done and what it will involve.

Please take the time to read the following information and feel free to ask for more information. We would like to stress that you do not have to accept this invitation and should only agree to take part if you want to.

Details of the study
The study is being conducted by a student investigator, Dr. Oddvar Ådnanes and supervised by Ms Selina Wallis, Liverpool School of Tropical Medicine. This study is a partial fulfillment of the requirements for completion of the Masters in Public Health degree. Ethical approval has been sought from the University of Liverpool and the Regional Committees for Medical and Health Research Ethics in Norway,prior to the start of this research.

Little is known about KAP of GPs in relation to TB in Norway, and the main purpose of this study is to establish baseline information on this topic. Furthermore the study will provide data that can inform future development of educational programmes on TB in Norway. Eastern Norway is chosen as research setting since the majority of new TB cases are detected here.

Details of participation
You will be asked to complete this online questionnaire after reviewing this information sheet. It will take you around 10 minutes to complete the survey and your online response will be considered as your consent. Participation is voluntary and all respondents are free to withdraw at anytime without explanation and without incurring any disadvantages. There is no known risk in participating in this study. There is no cost or payment to respondents for participation and no benefits awarded.

Confidentiality
All questionnaires will be anonymous and no personally identifying information will be collected. Data that is published or shared will be in aggregate so individual responses cannot be traced. Surveys will be completed here online and Dr. Ådnanes will store data securely on a password-protected account. After five years, all data will be destroyed.

Public access to results
After completion of studies with the University of Liverpool, analysis of the results in aggregate will be disseminated to relevant health authorities in Norway and on file in the library at the University of Liverpool.

Notification of problems
If you experience problems, or are unsatisfied with the conduct of this study, please feel free to let us know by contacting Ms Selina Wallis, supervisor, by email at: selina.wallis@my.ohecampus.com, and we will assist you. If you remain unsatisfied or have a complaint, which you feel cannot come to us, you should contact the university's research participant advocate at liverpoolethics@ohecampus.com or at 612-312-1210. If you have other questions you can contact Oddvar Aadnanes, telephone +4747731169 or email oddvar@psmail.net.

Please keep a copy of this document for your own record.

Thank you for reading this.

*1. Informed consent

- Yes. I would like to participate in this survey
- No. I dont want to participate in this survey

***2. What is your gender?**

- Male
- Female

***3. How old are you?**

- Under 30 years
- 31 – 40 years
- 41 – 50 years
- 51 – 60 years
- Over 60 years

***4. Are you a specialist in general practice?**

- Yes
- No

***5. How many patients do you have in your practice?**

- < 500
- 501 –900
- 901 – 1200
- 1201 – 1500
- > 1500

***6. How long have you been working in general practice?**

- < 1 year
- 1 – 4 years
- 5 – 9 years
- 10 – 14 years
- ≥ 15 years

***7. Do you work in a municipality with an asylum center?**

- Yes
- No

***8. Estimate, without going through the records, the number of patients you have diagnosed with tuberculosis and latent tuberculosis in your practice over the last three years?**

- ◯ 0 cases
- ◯ 1 – 2 cases
- ◯ 3 – 4 cases
- ◯ 5 – 6 cases
- ◯ 7 – 8 cases
- ◯ 9 – 10 cases
- ◯ > 10 cases

***9. In the past 12 months, have you attended a lecture/ seminar/ workshop on tuberculosis (TB)?**

- ◯ Yes
- ◯ No

***10. What are the main symptoms of pulmonary tuberculosis? Please check those you know.**

- ☐ Rash
- ☐ Cough
- ☐ Cough ≥3 weeks
- ☐ Cough with blood
- ☐ Severe headache
- ☐ Nausea
- ☐ Fever
- ☐ Chest pain
- ☐ Shortness of breath
- ☐ Swollen lymph nodes
- ☐ Weight loss
- ☐ Night sweats
- ☐ Back pain
- ☐ Reduced general condition
- ☐ Diarrhea

Other (please explain)

***11. Can a person who is infected with TB, go through life without getting sick? Please select only one answer.**

- ◯ Yes
- ◯ No
- ◯ I am not sure

***12. Does a positive Mantoux Test mean a definite TB infection? Please select only one answer**

- ◯ Yes
- ◯ No
- ◯ I am not sure

***13. Should a patient with positive IGRA test be referred to specialist for further examination for active or latent tuberculosis?**

- ◯ Yes
- ◯ No
- ◯ I am not sure

***14. Does a negative chest x-ray exclude TB infection?Please select only one answer.**

- ◯ Yes
- ◯ No
- ◯ I am not sure

***15. Does previous BCG vaccine ensure 100% protection against TB infection? Please select only one answer.**

- ◯ Yes
- ◯ No
- ◯ I am not sure

***16. Is a person with latent tuberculosis contagious? Please select only one answer.**

- ◯ Yes
- ◯ No
- ◯ I am not sure

***17. What is minimum duration of therapy for active TB? Please select only one answer.**

- ◯ 2 weeks
- ◯ 6 weeks
- ◯ 6 months
- ◯ 9 months
- ◯ I am not sure

***18. Which of these medications are part of the standard treatment of tuberculosis in Norway? Select the ones you know. Select " I am not sure" if the medications are unknown**

- [ ] Levofloxacin
- [ ] Etambutol
- [ ] Streptomycin
- [ ] Isoniazid
- [ ] Cycloserin
- [ ] Pyrazinamid
- [ ] Clarithromycin
- [ ] Rifampicin
- [ ] I am not sure

***19. What is DOT? Short description**

____

***20. An asymptomatic child from Somalia, age 10, arrived in Norway 3 months ago. Which statement is most appropriate?**

- ◯ No follow up needed as the child is asymptomatic
- ◯ Refer the child to IGRA-test and further to chest x-ray and specialist with positiv IGRA-test
- ◯ Check only BCG vaccination status. IGRA-test is not applicable for children
- ◯ The child should only be referred to chest x-ray
- ◯ I am not sure

***21. A 32 year-old asymptomatic woman who recently emigrated from Vietnam to Norway due to family reunion.Chest x-ray shows a right upper lobe cavitary lesion. Which statement is most appropriate?**

- ◯ Start preventive drug therapy for tuberculosis in communication with local TB coordinator
- ◯ Refer the patient to further testing with IGRA test and if negative no further follow up needed
- ◯ Treat the patient for a silent pneumonia with antibiotics
- ◯ Refer patient to local pulmonologist for further examinations
- ◯ Just wait and see. The patient is asymptomatic and this finding is common in Vietnam
- ◯ I am not sure

***22. A 26 year-old asymptomatic man has recently emigrated from Romania with a temporarily working contract with a local constructing company. Which statement is most appropriate?**

- ◯ None. He has emigrated from a European country
- ◯ Refer patient to IGRA test. No other tests are needed
- ◯ Refer patient for chest x-ray and if positive refer to local pulmonologist
- ◯ Provide patient and employer with a letter stating, “fit to work”
- ◯ Refer patient for Mantoux testing and BCG vaccination if the Mantoux test is negative.
- ◯ I am not sure

***23. One of your patients, an ethnic Norwegian teacher age 45, has recently returned back after working one year in a country with high incidence of TB. He has no symptoms and states he received BCG vaccine during childhood. Which statement is most appropriate?**

- ◯ None. Only health professionals returning from abroad need to be assessed
- ◯ None. He is BCG vaccinated
- ◯ Refer patient to further testing with Mantoux and chest x-ray
- ◯ Refer patient to IGRA testing with copy of the result to the local TB coordinator
- ◯ Refer patient to specialist for treatment against latent TB.
- ◯ I am not sure

***24. In your opinion, who are the persons most likely to become infected with TB in Norway? Please check all that apply.**

- ☐ Homeless persons
- ☐ Children under 5 years
- ☐ Senior Citizens
- ☐ People living with HIV/AIDS
- ☐ Health care workers returning from assignments abroad
- ☐ Health care workers treating a confirmed case
- ☐ Immigrants
- ☐ Family members of a confirmed case
- ☐ Prison Inmates
- ☐ Drug users
- ☐ Alcoholics

Others (Please explain)

***25. In your opinion, is TB a major public health threat in Norway? Please select only one answer.**

- ◯ Yes. Tuberculosis is already more than just a major threat
- ◯ Yes. Tuberculosis poses a serious threat to Norway
- ◯ No. Tuberculosis are well controlled so there is no major concern
- ◯ No.Tuberculosis is not even a small threat at this time
- ◯ I am not sure

***26. How can a person become infected with TB? Please select all that apply.**

- ☐ Through handshakes
- ☐ Through the air when a person with TB coughs or sneezes
- ☐ Through sharing dishes
- ☐ Through eating from the same plate
- ☐ Through contact with blood
- ☐ Through food and water
- ☐ Through touching items in public places (doorknobs)
- ☐ Through unprotected sex
- ☐ I am not sure

***27. Under what circumstances are health education messages on TB given to patients? Please check all that apply**

- ☐ World TB Day
- ☐ BCG Immunization
- ☐ General health promotion/ education messages delivered in clinical settings
- ☐ With suspected or confirmed cases only (i.e. no family members)
- ☐ With suspected cases and their families in a clinical setting
- ☐ With confirmed patients and their families in either a clinical or community setting
- ☐ Health education on TB is generally not done with patients

Other (Please explain):

***28. What is the primary diagnostic test that is usually requested in order to confirm or rule out a case of active pulmonary TB? Please select only one answer.**

- ◯ IGRA test
- ◯ Chest X-ray
- ◯ Mantoux test
- ◯ Sputum Smear Microscopy/ Culture
- ◯ Blood Culture
- ◯ I am not sure

***29. When can a TB patient be considered as noninfectious? Please select only one answer**

- ◯ Patient has received adequate TB treatment for a minimum of 2 weeks
- ◯ Patient has negative chest x-ray
- ◯ Patient has no cough
- ◯ Patient has completed the whole treatment
- ◯ Conversion of IGRA test from positive to negative
- ◯ I am not sure

***30. In your opinion, what is your role as GP when one of your patients is treated for TB? Please select only one answer.**

- ◯ My role is to be kept informed about the ongoing treatment and when appropriate I will be involved in the clinical monitoring of the patient under supervision by the specialist
- ◯ I have no role here. This is the responsibility of the specialist, TB coordinator and medical officers in the municipality
- ◯ I am not sure about my role in this case
