# Supplementary material for: A cross-sectional survey of the knowledge, attitudes and practices regarding tuberculosis among general practitioners working in municipalities with and without asylum centres in eastern Norway
Source: BMC Health Serv Res. 2018 Dec 20;18:987. doi: 10.1186/s12913-018-3792-4 (PMC6302494; doi:10.1186/s12913-018-3792-4)
Supplement: Supplementary file 2 — TB survey among Norwegian GPs survey questionnaire NOR: The survey questionnaire in Norwegian. (PDF 208 kb) [file 12913_2018_3792_MOESM2_ESM.pdf]

Spørreundersøkelse om tuberkulose

Informasjon om spørreundersøkelsen

Du er herved invitert til å delta i en spørreundersøkelse om kunnskap, holdninger og praksis til tuberkulose hos fastleger på Østlandet. Før du bestemmer deg om du ønsker å delta er det viktig at du vet hvorfor denne studien blir gjennomført og hva den innebærer.

Vennligst les gjennom denne informasjonen og be gjerne om mer informasjon dersom det er nødvendig. Vi ønsker å understreke at du ikke behøver å godta denne invitasjonen, og at du bare må delta dersom du har lyst.

Detaljer om studien
Studien blir utført av student Dr. Oddvar Ådnanes under veiledning av Selina Wallis, Liverpool School of Tropical Medicine. Denne studien er en del av kravene til et mastergrad studie i Public Health ved Universitetet i Liverpool. Etisk godkjennelse har blitt innhentet fra Universitetet i Liverpool samt Regional komité for medisinsk og helsefaglig forskningsetikk (REK) i Norge, referanse nummer 2014/1570.

Lite er kjent vedrørende kunnskaper om tuberkulose hos fastleger i Norge.
Formålet med denne studien er å beskrive hvordan dette ligger an og samtidig bidra med informasjon når fremtidige utdanningsprogrammer om tuberkulose skal utvikles. Studien vil bli gjennomført blant fastleger i 8 fylker på Østlandet da de fleste nye tuberkulosetilfellene blir oppdaget her.

Frivillig deltakelse
Du vil bli bedt om å svare på et nettbasert spørreskjema etter å ha gjennomgått denne informasjonen. Det vil ta deg ca. 10 minutter å svare på spørreskjemaet og din deltagelse i spørreundersøkelsen anses som et informert samtykke. Deltakelse er helt frivillig og du har muligheten til å trekke deg når som helst uten at det er behov for en forklaring. Det er ingen risiko ved å delta i denne studien og deltagere i studien mottar ingen betaling.

Konfidensialitet
Det nettbaserte spørreskjemaet besvares anonymt og ingen personopplysninger vil bli innhentet sammen med spørreskjemaet. Alle data vil bli behandlet konfidensielt og behandlet på en forsvarlig måte i henhold til Personopplysningsloven og retningslinjer fra Datatilsynet. Data som blir publisert eller delt med andre vil være anonymisert slik at ingen individuelle svar kan gjenkjennes. Veileder Selina Wallis og Dr. Ådnanes har taushetsplikt overfor alle opplysninger som samles inn. All data vil blir lagret sikkert og beskyttet med passord. All data vil bli slettet etter fem år.

Offentlig tilgang på resultatene
Resultatene vil bli publisert i en masteroppgave og lagret på biblioteket til Universitetet i Liverpool samt formidlet til relevante helsemyndigheter i Norge.

Spørsmål eller problemer
Hvis du opplever problemer eller er misfornøyd med hvordan denne studien blir gjennomført kan du ta kontakt med veileder Selina Wallis via e-post : selina.wallis@my.ohecampus.com. Hvis du fortsatt er misfornøyd eller har en klage som du føler du ikke kan dele med oss, kan du kontakte universitetet i Liverpool sin talsmann for etikk: liverpoolethics@ohecampus.com eller telefon 612-312-1210.Ved andre spørsmål kan du kontakte undertegnede: Oddvar Ådnanes, telefon 47731169, epost: oddvar@psmail.net

Takk for at du leser dette!

Spørreundersøkelse om tuberkulose

1. Informert samtykke

- Ja. Jeg ønsker å delta i denne studien
- Nei. Jeg ønsker ikke å delta i denne studien

*2. Hvilket kjønn er du?

- Mann
- Kvinne

*3. Hvor gammel er du?

- Under 30 år
- 31 – 40 år
- 41 – 50 år
- 51 – 60 år
- Over 60 år

*4. Er du spesialist i allmennmedisin?

- Ja
- Nei

*5. Hvor stor pasientliste har du?

- <500
- 501 –900
- 901 – 1200
- 1201 – 1500
- >1500

*6. Hvor lenge har du jobbet i en allmennpraksis?

- < 1 år
- 1 – 4 år
- 5 – 9 år
- 10 – 14 år
- ≥ 15 år

*7. Jobber du for tiden i en kommune med asylmottak?

- Ja
- Nei

***8. Anslå, uten å gå gjennom journalene, hvor mange av dine pasienter som har blitt diagnostisert med tuberkulose eller latent tuberkulose i praksisen din de tre siste årene?**

- ◯ 0 pasienter
- ◯ 1 – 2 pasienter
- ◯ 3 – 4 pasienter
- ◯ 5 – 6 pasienter
- ◯ 7 – 8 pasienter
- ◯ 9 – 10 pasienter
- ◯ > 10 pasienter

***9. Har du deltatt på kurs/seminarer/foredrag om tuberkulose de siste 12 månedene?**

- ◯ Ja
- ◯ Nei

***10. Hva er hovedsymptomene på lungetuberkulose? Kryss av for dem du vet om.**

- ☐ Utslett
- ☐ Hoste
- ☐ Hoste ≥3 uker
- ☐ Blodtilblandet oppspytt
- ☐ Sterk hodepine
- ☐ Kvalme
- ☐ Feber
- ☐ Brystsmerte
- ☐ Tungpust
- ☐ Hovne lymfeknuter
- ☐ Vekttap
- ☐ Nattesvette
- ☐ Ryggsmerter
- ☐ Nedsatt almenntilstand
- ☐ Diaré
- ☐ Andre (vennligst spesifiser)

Spørreundersøkelse om tuberkulose

***11. Kan en person som er smittet av tuberkulose, gå gjennom livet uten å bli syk? Vennligst velg bare ett svar.**

- ○ Ja
- ○ Nei
- ○ Vet ikke

***12. Er en positiv Mantoux test en sikker indikasjon på tuberkulosesmitte? Vennligst velg bare ett svar.**

- ○ Ja
- ○ Nei
- ○ Vet ikke

***13. Skal alle pasienter med positiv IGRA-test henvises til spesialist og utredes for aktiv eller latent tuberkulose? Vennligst velg bare ett svar.**

- ○ Ja
- ○ Nei
- ○ Vet ikke

***14. Vil et negativt lungerøntgen utelukke tuberkulosesmitte? Vennligst velg bare ett svar.**

- ○ Ja
- ○ Nei
- ○ Vet ikke

***15. Vil BCG-vaksinasjon beskytte 100% mot tuberkulose? Vennligst velg bare ett svar.**

- ○ Ja
- ○ Nei
- ○ Vet ikke

***16. Er en person med latent tuberkulose smittsom? Vennligst velg bare ett svar.**

- ○ Ja
- ○ Nei
- ○ Vet ikke

***17. Hva er minimum varighet for standard behandling av aktiv tuberkulose? Vennligst velg bare ett svar.**

- ◯ 2 uker
- ◯ 6 uker
- ◯ 6 måneder
- ◯ 9 måneder
- ◯ Vet ikke

***18. Hvilke av disse medikamentene inngår i standardbehandlingen av tuberkulose i Norge? Kryss av for dem du vet om. Kryss vet ikke dersom medikament navnene er ukjente.**

- ☐ Levofloxacin
- ☐ Etambutol
- ☐ Streptomycin
- ☐ Isoniazid
- ☐ Cycloserin
- ☐ Pyrazinamid
- ☐ Clarithromycin
- ☐ Rifampicin
- ☐ Vet ikke

***19. Hva er DOT? (Beskriv kort)**

***20. Et 10 år gammelt barn fra Somalia ankom Norge for 3 måneder siden.Barnet har ingen kjente symptomer eller plager. Hvilket utsagn passer best i dette tilfellet?**

- ◯ Oppfølging er ikke nødvendig siden barnet ikke har symptomer eller plager
- ◯ Barnet skal henvises til IGRA-test og videre til lungerøntgen og spesialist ved positiv IGRA test
- ◯ Kun vurdere BCG-vaksinasjonsstatus. IGRA test er ikke aktuelt hos barn.
- ◯ Barnet skal kun henvises til lungerøntgen
- ◯ Vet ikke

***21. En 32 år gammel vietnamesisk kvinne har nylig ankommet Norge grunnet familiegjenforening. Kvinnen har ingen symptomer. Lungerøntgen viser en veldefinert rundskygge med kaverne i høyre lunges overlapp. Hvilket utsagn passer best i dette tilfellet?**

- ◯ Starte forebyggende behandling for tuberkulose i nær dialog med den lokale tuberkulosekoordinatoren.
- ◯ Henvise pasienten til IGRA-test og ved negativt resultat avslutte oppfølgingen
- ◯ Starte behandling med antibiotika for antatt underliggende lungebetennelse
- ◯ Henvise pasienten til lokal lungespesialist for videre vurdering
- ◯ Vente å se. Pasienten har ingen symptomer og slike røntgenfunn er vanlige i Vietnam.
- ◯ Vet ikke

***22. En 26 år gammel mann fra Romania har nylig ankommet Norge med en midlertidig arbeidskontrakt med et lokalt byggefirma. Han føler seg frisk og har ingen symptomer. Hvilket utsagn passer best i dette tilfellet?**

- ◯ Ikke nødvendig med videre tiltak siden mannen kommer fra et land i Europa
- ◯ Henvise mannen til IGRA-test og ingen andre tester er nødvendige.
- ◯ Henvise mannen til lungerøntgen og videre henvisning til spesialist dersom det er positive forandringer på lungerøntgen
- ◯ Gi mannen en helseattest som uttrykker at mannen er arbeidsfør
- ◯ Henvise mannen til Mantoux-test og påfølgende BCG-vaksinering dersom testen er negativ
- ◯ Vet ikke

***23. En av dine pasienter, en etnisk norsk lærer på 45 år, har nylig kommet hjem etter å ha jobbet et år i et land med høy forekomst av tuberkulose. Han har ingen symptomer og sier han ble BCG-vaksinert i barndommen. Hvilket utsagn passer best i dette tilfellet?**

- ◯ Ikke nødvendig med videre tiltak siden mannen ikke er helsepersonell
- ◯ Ikke nødvendig med videre tiltak siden mannen er BCG-vaksinert.
- ◯ Henvise mannen kun til lungerøntgen og dersom det er negativt avslutte oppfølgingen
- ◯ Henvise mannen til IGRA-test og lungerøntgen og ved positiv prøve videre henvisning til spesialist
- ◯ Henvise mannen til spesialist for vurdering av medikamentell behandling mot latent tuberkulose.
- ◯ Vet ikke

***24. Hvilken gruppe tror du er mest utsatt for å utvikle tuberkulose i Norge idag? Kryss av for alle som passer.**

- [ ] Hjemløse personer
- [ ] Barn under 5 år
- [ ] Pensjonister
- [ ] Mennesker med nedsatt immunforsvar f.eks HIV/AIDS
- [ ] Helsepersonell som returnerer fra oppdrag i utlandet
- [ ] Helsepersonell som behandler pasienter med tuberkulose
- [ ] Innvandrere
- [ ] Familiemedlemmer av personer med tuberkulose
- [ ] Innsatte i fengsel
- [ ] Rusmisbrukere
- [ ] Andre (vennligst beskriv)

***25. Utgjør tuberkulose etter din mening en alvorlig trussel mot folkehelsen i Norge? Vennligst velg bare ett svar.**

- ○ Ja. Tuberkulose er allerede mer enn en alvorlig trussel.
- ○ Ja. Tuberkulose utgjør en alvorlig trussel i Norge.
- ○ Nei. Tuberkulose er godt kontrollert så det er lite grunn til bekymring.
- ○ Nei. Tuberkulose utgjør nå ikke engang en liten trussel.
- ○ Vet ikke.

***26. Hvordan kan en person bli smittet av tuberkulose? Kryss av for alle som passer.**

- [ ] Ved håndtrykk
- [ ] Gjennom luft når en person med tuberkulose hoster eller nyser
- [ ] Ved å dele bestikk
- [ ] Ved å spise fra samme tallerken
- [ ] Ved kontakt med blod
- [ ] Gjennom mat og drikke
- [ ] Ved berøring av kontaktpunkter på offentlige steder (eks dørhåndtak)
- [ ] Ved ubeskyttet sex
- [ ] Vet ikke

***27. Når har du formidlet helseopplysning om tuberkulose til dine pasienter? Kryss av for alle som passer.**

- [ ] I forbindelse med Verdens Tuberkulose Dag
- [ ] I forbindelse med BCG vaksinasjon
- [ ] På kontoret i forbindelse med formidling av allmenn helseopplysning
- [ ] På kontoret til mulige og bekreftede tilfeller (dvs. Ikke til deres familiemedlemmer)
- [ ] På kontoret til mulige tilfeller og deres familier
- [ ] På kontoret og i nærmiljøet til mulige tilfeller og deres familier
- [ ] Pleier ikke å formidle helseopplysning om tuberkulose til pasientene mine
- [ ] Andre anledninger (vennligst spesifiser)

***28. Hva er etter din mening den primære diagnostiske testen som blir brukt til å bekrefte eller avkrefte aktiv lungetuberkulose? Vennligst velg bare ett svar.**

- ○ IGRA-test
- ○ Lungerøntgen
- ○ Mantoux-test
- ○ Mikrobiologisk undersøkelse av ekspektorat
- ○ Blodkultur
- ○ Vet ikke

***29. Hvilket utsagn passer best på en pasient med lungetuberkulose med følsomme bakterier som ikke lenger anses som smittsom? Vennligst velg bare ett svar.**

- ○ Pasienten har fått minst 2 uker med medikamentell behandling mot lungetuberkulose
- ○ Pasienten har et negativ lungerøntgen
- ○ Pasienten hoster ikke lengre
- ○ Pasienten må ha fullført all medikamentell behandling fullstendig
- ○ Konvertering av IGRA-test fra positiv til negativ
- ○ Vet ikke

***30. Hvilket utsagn passer best for å beskrive din rolle som fastlege når en av pasientene dine blir behandlet for tuberkulose? Vennligst velg bare ett svar.**

- ○ Min rolle er å bli holdt orientert om den pågående behandlingen og evt delta i samarbeidsgruppe dersom pasienten ønsker dette
- ○ Jeg har ingen rolle her siden dette blir ivaretatt av ansvarlig spesialist, tuberkulose koordinator og kommunehelsetjenesten.
- ○ Jeg er usikker på min rolle her
